# Supplementary material for: Renal function in a cohort of HIV-infected patients initiating antiretroviral therapy in an outpatient setting in Ethiopia
Source: PLoS One. 2021 Jan 22;16(1):e0245500. doi: 10.1371/journal.pone.0245500 (PMC7822244; doi:10.1371/journal.pone.0245500)
Supplement: S1 Table — (DOCX) [file pone.0245500.s002.docx]

**S Table 1: Baseline characteristics of patients included and excluded from the analysis**

| **Characteristics** | | **Included**  **(N = 353)** | **Excluded**  **(N = 295)** | ***P–value*** |
| --- | --- | --- | --- | --- |
| Age (year), mean ± SD | | 37.5 ± 9.7 | 37.3 ± 10.3 | 0.713 |
| Sex, n (%) |  |  |  | 0.781 |
|  | Male | 145 (41.1) | 118 (40.0) |  |
|  | Female | 208 (58.9) | 177 (60.0) |  |
| WHO clinical stage, n (%) |  |  |  | 0.627 |
|  | 1/2 | 288 (81.6) | 245 (83.1) |  |
|  | 3/4 | 65 (18.4) | 50 (16.9) |  |
| CD4 count (Cells/mm^3^), mean ± SD | | 406.5 ± 274 | 398.5 ± 217 | 0.690 |
| Diabetes or hypertension, n (%) |  |  |  | 0.257 |
|  | Yes | 27 (7.6) | 16 (5.4) |  |
| Hemoglobin (g/dl), mean ± SD | | 13.6 ± 2.9 | 13.4 ± 3.1 | 0.447 |
| Total cholesterol (mg/dl), mean ± SD | | 177.4 ± 48.5 | 173.3 ± 52.7 | 0.549 |
