# Supplementary material for: Renal function in a cohort of HIV-infected patients initiating antiretroviral therapy in an outpatient setting in Ethiopia
Source: PLoS One. 2021 Jan 22;16(1):e0245500. doi: 10.1371/journal.pone.0245500 (PMC7822244; doi:10.1371/journal.pone.0245500)

PLOS ONE

Renal function in a cohort of HIV-infected patients initiating antiretroviral therapy in an outpatient setting in Ethiopia

--Manuscript Draft--

**Manuscript Number:**	PONE-D-20-19005
**Article Type:**	Research Article
**Full Title:**	Renal function in a cohort of HIV-infected patients initiating antiretroviral therapy in an outpatient setting in Ethiopia
**Short Title:**	Renal function in a cohort of HIV-infected patients initiating ART
**Corresponding Author:**	Temesgen Fiseha<br>Wollo University<br>Wollo, ETHIOPIA
**Keywords:**	Renal function, HIV Patients, ART
**Abstract:**	Aim<br><br>To evaluate the prevalence and associated factors of abnormal renal function among Ethiopian HIV-infected patients at baseline prior to initiation of antiretroviral therapy (ART) and during follow-up.<br><br>Methods<br><br>We conducted a retrospective cohort study of HIV infected patients who initiated ART at the outpatient ART clinic of Mehal Meda Hospital of North Shewa, Ethiopia from January 2012 to August 2018. Demographic and clinical data were abstracted from the medical records of patients. Renal function was assessed by estimated glomerular filtration rate (eGFR) calculated using the Modification of Diet in Renal Disease (MDRD) equation. Univariate and multivariate analysis were conducted to determine the factors associated with abnormal renal function at baseline and during follow-up.<br><br>Results<br><br>Among 353 patients, 70 (19.8%) had baseline eGFR <60 ml/min/1.73m2 and 102 (28.9%) had eGFR = 60–89.9 ml/min/1.73m2. Factors associated with baseline renal impairment (eGFR <60 ml/min/1.73m2) included female sex (AOR = 3.52, CI 1.75–7.09), CD4 count < 200 cells/mm3 (AOR = 2.75, CI 1.40–5.42), BMI < 25 Kg/m2 (AOR = 3.04, CI 1.15–8.92), low hemoglobin (AOR = 2.19, CI 1.16–4.09) and high total cholesterol (AOR = 3.15, CI 1.68–5.92). After a median of 3.0 years of ART, the mean eGFR declined from 112.9 ± 81.2 ml/min/1.73m2 at baseline to 93.9 ± 60.6 ml/min/1.73m2 (P < 0.001). The prevalence of renal impairment increased from 19.8% at baseline to 22.1% during follow-up. Of 181 patients with baseline normal renal function, 49.7% experienced some degree of renal impairment. Older age (AOR = 3.85, 95% CI 2.03–7.31), female sex (AOR = 4.18, 95% CI 2.08–8.40), low baseline CD4 (AOR = 2.41, 95% CI 1.24–4.69), low current CD4 count (AOR = 2.32, 95% CI 1.15–4.68), high BMI (AOR = 2.91, 95% CI 1.49–5.71), and low hemoglobin (AOR = 3.38, 95% CI 2.00–7.46) were the factors associated with renal impairment during follow-up.<br><br>Conclusion<br><br>Impaired renal function was common in HIV-infected patients initiating ART in an outpatient setting in Ethiopia, and there appears to be a high prevalence of renal impairment after a median ART follow-up of 3 years. There is a need for assessment of renal function at baseline before ART initiation and regular monitoring of renal function for patients with HIV during follow-up.
**Order of Authors:**	Temesgen Fiseha
	Angesom Gebreweld

Powered by Editorial Manager® and ProduXion Manager® from Aries Systems Corporation

Additional Information:	
Question	Response
**Financial Disclosure**<br><br>Enter a financial disclosure statement that describes the sources of funding for the work included in this submission. Review the submission guidelines for detailed requirements. View published research articles from *PLOS ONE* for specific examples.<br><br>This statement is required for submission and **will appear in the published article** if the submission is accepted. Please make sure it is accurate.<br><br>**Unfunded studies**<br>Enter: *The author(s) received no specific funding for this work.*<br><br>**Funded studies**<br>Enter a statement with the following details:<br>• Initials of the authors who received each award<br>• Grant numbers awarded to each author<br>• The full name of each funder<br>• URL of each funder website<br>• Did the sponsors or funders play any role in the study design, data collection and analysis, decision to publish, or preparation of the manuscript?<br>• **NO** - Include this sentence at the end of your statement: *The funders had no role in study design, data collection and analysis, decision to publish, or preparation of the manuscript.*<br>• **YES** - Specify the role(s) played.<br><br>* typeset	The author(s) received no specific funding for this work.
**Competing Interests**<br><br>Use the instructions below to enter a competing interest statement for this submission. On behalf of all authors, disclose any competing interests that could be perceived to bias this work—acknowledging all financial support and any other relevant financial or non-financial competing interests.	The authors have declared that no competing interests exist

Powered by Editorial Manager® and ProduXion Manager® from Aries Systems Corporation

This statement **will appear in the published article** if the submission is accepted. Please make sure it is accurate. View published research articles from *PLOS ONE* for specific examples.<br><br>**NO authors have competing interests**<br><br>Enter: *The authors have declared that no competing interests exist.*<br><br>**Authors with competing interests**<br><br>Enter competing interest details beginning with this statement:<br><br>*I have read the journal's policy and the authors of this manuscript have the following competing interests: [insert competing interests here]*<br><br>* typeset	
**Ethics Statement**<br><br>Enter an ethics statement for this submission. This statement is required if the study involved:<br><br>• Human participants<br>• Human specimens or tissue<br>• Vertebrate animals or cephalopods<br>• Vertebrate embryos or tissues<br>• Field research<br><br>Write "N/A" if the submission does not require an ethics statement.<br><br>General guidance is provided below. Consult the submission guidelines for detailed instructions. **Make sure that all information entered here is included in the Methods section of the manuscript.**	N/A

**Format for specific study types**<br><br>**Human Subject Research (involving human participants and/or tissue)**<br>• Give the name of the institutional review board or ethics committee that approved the study<br>• Include the approval number and/or a statement indicating approval of this research<br>• Indicate the form of consent obtained (written/oral) or the reason that consent was not obtained (e.g. the data were analyzed anonymously)<br><br>**Animal Research (involving vertebrate animals, embryos or tissues)**<br>• Provide the name of the Institutional Animal Care and Use Committee (IACUC) or other relevant ethics board that reviewed the study protocol, and indicate whether they approved this research or granted a formal waiver of ethical approval<br>• Include an approval number if one was obtained<br>• If the study involved *non-human primates*, add *additional details* about animal welfare and steps taken to ameliorate suffering<br>• If anesthesia, euthanasia, or any kind of animal sacrifice is part of the study, include briefly which substances and/or methods were applied<br><br>**Field Research**<br><br>Include the following details if this study involves the collection of plant, animal, or other materials from a natural setting:<br>• Field permit number<br>• Name of the institution or relevant body that granted permission	
**Data Availability**<br><br>Authors are required to make all data underlying the findings described fully available, without restriction, and from the time of publication. PLOS allows rare exceptions to address legal and ethical concerns. See the PLOS Data Policy and FAQ for detailed information.	Yes - all data are fully available without restriction

A Data Availability Statement describing where the data can be found is required at submission. Your answers to this question constitute the Data Availability Statement and **will be published in the article**, if accepted.<br><br>**Important:** Stating 'data available on request from the author' is not sufficient. If your data are only available upon request, select 'No' for the first question and explain your exceptional situation in the text box.<br><br>Do the authors confirm that all data underlying the findings described in their manuscript are fully available without restriction?	
**Describe where the data may be found in full sentences. If you are copying our sample text, replace any instances of XXX with the appropriate details.**<br><br>• If the data are **held or will be held in a public repository**, include URLs, accession numbers or DOIs. If this information will only be available after acceptance, indicate this by ticking the box below. For example: *All XXX files are available from the XXX database (accession number(s) XXX, XXX.).*<br>• If the data are all contained **within the manuscript and/or Supporting Information files**, enter the following: *All relevant data are within the manuscript and its Supporting Information files.*<br>• If neither of these applies but you are able to provide **details of access elsewhere**, with or without limitations, please do so. For example:<br><br>*Data cannot be shared publicly because of [XXX]. Data are available from the XXX Institutional Data Access / Ethics Committee (contact via XXX) for researchers who meet the criteria for access to confidential data.*<br><br>*The data underlying the results presented in the study are available from (include the name of the third party*	All relevant data are within the manuscript and its Supporting Information files.

and contact information or URL).<br>• This text is appropriate if the data are owned by a third party and authors do not have permission to share the data.<br><br>* typeset	
Additional data availability information:	

Powered by Editorial Manager® and ProduXion Manager® from Aries Systems Corporation

Click here to download Manuscript Befor -After eGFR (2).docx

Renal function in a cohort of HIV-infected patients initiating antiretroviral therapy in an outpatient setting in Ethiopia

Temesgen Fiseha[1*] and Angesom Gebreweld[2]

Address:

[1] Department of Clinical Laboratory Science, College of Medicine and Health Sciences, Wollo University, Dessie, Ethiopia

[2] Department of Medical Laboratory Science, College of Health Sciences, Mekelle University, Mekelle, Ethiopia

TF-temafiseha@gmail.com, AG-afsaha@gmail.com

corresponding author

Abstract

Aim

To evaluate the prevalence and associated factors of abnormal renal function among Ethiopian HIV-infected patients at baseline prior to initiation of antiretroviral therapy (ART) and during follow-up.

Methods

We conducted a retrospective cohort study of HIV infected patients who initiated ART at the outpatient ART clinic of Mehal Meda Hospital of North Shewa, Ethiopia from January 2012 to August 2018. Demographic and clinical data were abstracted from the medical records of patients. Renal function was assessed by estimated glomerular filtration rate (eGFR) calculated using the Modification of Diet in Renal Disease (MDRD) equation. Univariate and multivariate analysis were conducted to determine the factors associated with abnormal renal function at baseline and during follow-up.

Results

Among 353 patients, 70 (19.8%) had baseline eGFR <60 ml/min/1.73m^2 and 102 (28.9%) had eGFR = 60–89.9 ml/min/1.73m^2. Factors associated with baseline renal impairment (eGFR <60 ml/min/1.73m^2) included female sex (AOR = 3.52, CI 1.75–7.09), CD4 count < 200 cells/mm^3 (AOR = 2.75, CI 1.40–5.42), BMI < 25 Kg/m^2 (AOR = 3.04, CI 1.15–8.92), low hemoglobin (AOR = 2.19, CI 1.16–4.09) and high total cholesterol (AOR = 3.15, CI 1.68–5.92). After a median of 3.0 years of ART, the mean eGFR declined from 112.9 ± 81.2 ml/min/1.73m^2 at baseline to 93.9 ± 60.6 ml/min/1.73m^2 ($P < 0.001$). The prevalence of renal impairment increased from 19.8% at baseline to 22.1% during follow-up. Of 181 patients with baseline normal renal function, 49.7% experienced some degree of renal impairment. Older age (AOR = 3.85, 95% CI 2.03–7.31), female sex (AOR = 4.18, 95% CI 2.08–8.40), low baseline CD4 (AOR = 2.41, 95% CI 1.24–4.69), low current CD4 count (AOR = 2.32, 95% CI 1.15–4.68), high BMI (AOR = 2.91, 95% CI 1.49–5.71), and low hemoglobin (AOR = 3.38, 95% CI 2.00–7.46) were the factors associated with renal impairment during follow-up.

Conclusion

Impaired renal function was common in HIV-infected patients initiating ART in an outpatient setting in Ethiopia, and there appears to be a high prevalence of renal impairment after a median ART follow-up of 3 years. There is a need for assessment of renal function at baseline before ART initiation and regular monitoring of renal function for patients with HIV during follow-up.

Introduction

Impaired renal function is a common complication of HIV infection in both the pre-antiretroviral therapy (ART) and ART periods, and is associated with increased risk of HIV disease progression, AIDS- and non-AIDS-related events, cardiovascular disease and mortality (1–5). In patients with HIV, impaired renal function has been shown to be a risk factor for drug-related adverse events, acute renal failure, hospital admissions and progression to end-stage renal disease requiring renal replacement therapies in the form of dialysis or transplantation, which may impact quality of life and survival (5–8). As a consequence, assessment of renal function is recommended for all HIV-infected individuals at baseline prior to initiation of ART and during follow-up (9).

Renal impairment during the pre-ART period was largely a result of HIV-associated renal disease, which is a leading cause of chronic kidney disease and end-stage renal disease, especially in persons of African descent and is caused by direct injury to the kidneys by the HIV (10,11). Although the incidence of HIV-associated renal impairment has decreased in the recent ART period, the proportion of renal impairment has increased due to increasing burden of comorbid renal impairment risk factors such as diabetes and hypertension in this aging population (12–14). The increase in the incidence of renal impairment could also be related to the long-term exposure to potentially nephrotoxic antiretrovirals, such as tenofovir, ritonavir-boosted atazanavir, and ritonavir-boosted lopinavir, or to the effects of HIV infection itself, pre-existing renal impairment and of co-infections (15–18).

Renal impairment was seen in up to 24% of patients at baseline prior to ART initiation (19) and there are numerous studies demonstrating renal function improvement after initiation of ART (20,21). Others have reported no significant improvement and/or progressive loos of renal function during follow-up (22–24). Given the high burden of HIV and the introduction of the "test-and treat" strategy in sub-Saharan Africa, an understanding of the prevalence and associated factors of renal impairment before and during ART follow-up is imperative to guide strategies in the management and clinical care of this population. The aim of our study was therefore to evaluate the prevalence and associated risk factors of abnormal renal function at baseline and during follow-up and to describe the impact of ART on renal function in a cohort of HIV-infected patients initiating ART in an outpatient setting in Ethiopia.

Methods

Study design and population

This was a retrospective cohort study of HIV infected patients who initiated ART at the outpatient ART clinic of Mehal Meda Hospital of North Shewa, Ethiopia from January 2012 to August 2018. A total of 353 HIV-infected patients on ART follow-up were included in the study. The inclusion criteria were age older than 18 years, receipt of ART for 6 months or more, and measured serum creatinine results at the baseline prior to initiation of ART and during follow-up (after the initiation of ART). Patients with missing data for essential variables, pregnant, and end-stage renal disease on dialysis patients were excluded from the study analysis. The study protocol was approved by the Institutional Review Board of College of Medicine and Health Sciences, Wollo University.

Data collection

Data were abstracted from the medical records of the patients. The data obtained included demographics (e.g., age, sex, residence, education, height, and body weight), history of diabetes and/or hypertension and clinical information (e.g., CD4 cell count, World Health Organization (WHO) HIV/AIDS clinical stage, hemoglobin test results, serum total cholesterol and serum creatinine, duration of ART use, and types of ART regimens). Serum creatinine was measured and recorded at baseline prior to ART initiation, at 3 and 6 months after initiating therapy, and annually thereafter. After initiation of therapy, if a patient had multiple documented serum creatinine results, only the most recent result was utilized for the analysis. Renal function was assessed by estimated glomerular filtration rate (eGFR) calculated using the Modification of Diet in Renal Disease (MDRD) equation (25), and categorized into 3 categories (eGFR ≥90, 60-89.9 and < 60 ml/min/1.73 m^2) according to the National Kidney Foundation criteria (26). Patients were defined as having renal impairment at baseline and during follow up if eGFR values were < 60 ml/min/1.73 m^2.

Statistical Analysis

Patient data were entered into an "EpiData version 3.1" and analyzed using SPSS version 20 software (SPSS Inc., Chicago, IL, USA). Categorical variables were summarized by frequencies

and percentages, and continuous variables were summarized by mean ± standard deviation (SD). Univariate analyses to assess factors associated with renal impairment at baseline and during follow-up were tested using Chi-square test and student's t-test, where appropriate. Variables that were found to be significant in univariate analysis (*P* < 0.25) were included in the multivariate backwards stepwise logistic regression model to identify factors independently associated with renal impairment. *P*-values < 0.05 were considered significant.

Results

A total of 353 HIV-infected patients on ART with serum creatinine data available at the baseline and during follow-up were included in the study. The baseline demographic and clinical characteristics of the patients are summarized in Table 1. Mean (± SD) age was 37.5 ± 9.7 years and 58.9% were females. Mean BMI was 19.6 ± 4.1 kg/m^2; and only 16.7% were overweight/obese (BMI ≥ 25 kg/m^2). The mean baseline CD4 count was 406.5 ± 274 cells/mm^3, with 25.5% of patients presenting with a CD4 count of <200 cells/mm^3. An advanced HIV disease stage (WHO stage 3/4) was present in 18.4% of patients at baseline. The mean hemoglobin, total cholesterol and serum creatinine were 13.6 ± 2.9 g/dl, 177.4 ± 48.5 mg/dl and 1.06 ± 0.48 mg/dl respectively. Twenty-seven (7.6%) patients had a history of diabetes or hypertension at baseline.

Table 1: Baseline characteristics of the study patients (n = 353)

**Characteristics**		
Age (year), mean ± SD		37.5 ± 9.7
Age group, n (%)		
	18-30 years	103 (29.2)
	31-50 years	228 (64.6)
	> 50 years	22 (6.2)
Sex, n (%)		
	Male	145 (41.1)
	Female	208 (58.9)
Residence, n (%)		
	Urban	227 (64.3)
	Rural	126 (35.7)
Education, n (%)		
	< High school	291 (82.4)
	≥ High school	62 (17.6)
WHO clinical stage, n (%)		
	1/2	288 (81.6)
	3/4	65 (18.4)
CD4 count (Cells/mm^3), mean ± SD		406.5 ± 274
CD4 category, n (%)		
	< 200 Cells/mm^3	90 (25.5)
	200-350 Cells/mm^3	85 (24.1)
	> 350 Cells/mm^3	178 (50.4)
Body mass index (kg/m^2), mean ± SD		19.6 ± 4.1
Diabetes or hypertension, n (%)		
	Yes	27 (7.6)
	No	326 (92.4)
Hemoglobin (g/dl), mean ± SD		13.6 ± 2.9
Total cholesterol (mg/dl), mean ± SD		177.4 ± 48.5
Serum Creatinine (mg/dl), mean ± SD		1.06 ± 0.48

Renal function at baseline

The mean baseline eGFR of the study patients was 112.9 ± 81.2 ml/min/1.73m^2. A total of 70 (19.8%) patients had baseline eGFR <60 ml/min/1.73m^2, 102 (28.9%) had eGFR = 60–89.9 ml/min/1.73m^2, and 181 (51.3%) had eGFR ≥ 90 ml/min/1.73m^2. In univariate analysis, age over 50 years (OR = 3.29, 95% CI 1.57–6.90) and female sex (OR = 2.82, CI 1.54–5.18) were associated with renal impairment (eGFR < 60 ml/min/1.73 m^2) at baseline before ART initiation. Low CD4 count, low BMI, low hemoglobin, and high total cholesterol were also associated with renal impairment. No association was found with residence, education, WHO clinical disease stage and comorbid diabetes or hypertension (Table 2). Multivariable logistic regression analysis identified female sex (AOR = 3.52, CI 1.75–7.09; P < 0.001), CD4 count < 200 cells/mm^3 (AOR = 2.75, CI 1.40–5.42; P = 0.004), BMI < 25 Kg/m^2 (AOR = 3.04, CI 1.15–8.92; P = 0.030), low hemoglobin < 12 g/dl (AOR = 2.19, CI 1.16–4.09; P = 0.015), and high total cholesterol ≥ 200 mg/dl (AOR = 3.15, CI 1.68–5.92; P < 0.001) as risk factors for baseline renal impairment.

Renal function on ART follow up

The median duration on ART was 3.0 years (interquartile range [IQR], 1.5–5.4 years) and 221 (62.6%) patients were on non-TDF based ART regimens. The mean CD4 count was an average of 96.2 cells/mm^3 higher than baseline (502.8 ± 289 vs 406.5 ± 274 cells/mm^3, P < 0.001). The mean eGFR declined from 112.9 ± 81.2 ml/min/1.73 m^2 at baseline to 93.9 ± 60.6 ml/min/1.73m^2 after a median ART follow-up of 3 years (P < 0.001). The prevalence of renal impairment (eGFR <60 ml/min/1.73 m^2) increased from 19.8% at baseline to 22.1% after a median of 3 years on ART. The proportion of patients with eGFR = 60–89.9 ml/min/1.73m^2 increased by 10.2% after ART. Of 70 patients with eGFR <60 ml/min/1.73m^2 at baseline, 40% still had eGFR <60 ml/min/1.73m^2, 40% experienced eGFR = 60–90 ml/min/1.73 m^2 and 20.0% recovered to eGFR ≥90 ml/min/1.73 m^2 after a median of 3 years of ART. For the 102 patients who had eGFR 60–90 ml/min/1.73 m^2 at baseline, 27.5% experienced a decreased in eGFR to <60 ml/min/1.73 m^2 and 31.3% improved to normal eGFR >90 ml/min/1.73m ^{2} after a median of 3 years on ART. Among 181 patients with baseline normal eGFR ≥90 ml/min/1.73 m^2, 12.1% experienced a decrease in eGFR to <60 ml/min/1.73m^2 and 37.6% experienced eGFR = 60–89.9 ml/min/1.73m^2 after a median treatment duration of 3 years (Fig 1).

Fig 1. Distribution of eGFR categories of HIV-infected patients on ART stratified by baseline eGFR

The univariate analysis demonstrated that older age (OR = 3.95, 95% CI 2.30–6.80), female sex (OR = 3.77, 95% CI 2.04–6.94), low baseline CD4 (OR = 2.42, 95% CI 1.41–4.13), current CD4 count (OR = 3.11, 95% CI 1.75–5.52), duration on ART (OR = 1.74, 95% CI 1.04–2.90), high BMI (OR = 3.38, 95% CI 1.92–5.95), low hemoglobin < 12 g/dl (OR = 3.67, 95% CI 2.09–6.47), and baseline renal impairment (OR = 3.11, 95% CI 1.76–5.48) were associated with renal impairment during ART follow-up (Table 3). We did not find any association between renal impairment and residence, education, WHO clinical disease stage, or treatment with a TDF-based regimen. In the multivariable analysis, older age (AOR = 3.85, 95% CI 2.03–7.31; *P* < 0.001), female sex (AOR = 4.18, 95% CI 2.08–8.40; *P* < 0.001), low baseline CD4 (AOR = 2.41, 95% CI 1.24–4.69; *P* = 0.009), low current CD4 count (AOR = 2.32, 95% CI 1.15–4.68; *P* = 0.018), high BMI (AOR = 2.91, 95% CI 1.49–5.71; *P* = 0.002), and low hemoglobin < 12 g/dl (AOR = 3.38, 95% CI 2.00–7.46; *P* = 0.001) remained significantly associated with renal impairment.

Table 2: Factors associated with renal impairment (eGFR < 60 ml/min/1.73 m^2) at baseline

Characteristic	eGFR < 60 ml/min/1.73m^2		Crude OR (95% CI)	*P*-value	Adjusted OR (95% CI)	*P*-value
	Yes (n = 70)	No (n = 283)				
Age (years)						
< 50	56 (17.6)	263 (82.4)	1	0.001	1	0.174
≥ 50	14 (41.2)	20 (58.8)	3.29 (1.57–6.90)		1.75 (0.71–4.30)	
Sex						
Male	16 (11.0)	129 (89.0)	1	0.001	1	0.001
Female	54 (26.0)	154 (74.0)	2.82 (1.54–5.18)		3.52 (1.75–7.09)	
Residence						
Urban	48 (21.1)	179 (78.9)	1.27 (0.72–2.23)	0.405		
Rural	22 (17.5)	104 (82.5)	1			
Education						
< High school	63 (21.6)	228 (78.4)	2.17 (0.94–5.00)	0.063	1.81 (0.72–4.57)	0.208
≥ High school	7 (11.3)	55 (88.7)	1			
CD4 count (cells/mm3)						
< 200	28 (31.4)	62 (68.9)	2.53 (1.38–4.63)	0.003	2.75 (1.40–5.42)	0.004
200–350	15 (17.6)	70 (82.4)	1.20 (0.60–2.39)	0.608	1.36 (0.64–2.89)	0.432

> 350	27 (15.2)	151 (84.8)	1		1	
WHO Clinical Stage						
1/2	56 (19.4)	232 (80.6)	1	0.702		
3/4	14 (21.5)	51 (78.5)	0.68 (0.46–1.70)			
Body mass index (Kg/m^2)						
< 25	65 (22.1)	229 (77.9)	3.07 (1.18–7.98)	0.017	3.04 (1.15–8.92)	0.030
≥ 25	5 (8.5)	54 (91.5)	1		1	
Hemoglobin (g/dl)						
< 12	27 (34.2)	52 (65.8)	2.79 (1.58–4.92)	< 0.001	2.19 (1.16–4.09)	0.015
≥ 12	43 (15.7)	231 (84.3)	1		1	
Diabetes or hypertension						
Yes	9 (33.3)	18 (66.7)	2.17 (0.93–5.07)	0.067	1.88 (0.76–4.67)	0.226
No	61 (18.7)	265 (81.3)	1		1	
Total cholesterol (mg/dl)						
< 200	43 (15.6)	233 (84.4)	1	< 0.001	1	<0.001
≥ 200	27 (35.1)	50 (64.9)	2.93 (1.66–5.17)		3.15 (1.68–5.92)	

Table 3: Factors associated with renal impairment (eGFR < 60 ml/min/1.73 m^2) during ART follow-up

**Characteristic**	**eGFR <60 ml/min/1.73m^2**		**Crude OR (95% CI)**	***P*-value**	**Adjusted OR (95% CI)**	***P*-value**
	**Yes (n = 78)**	**No (n = 275)**				
Age (years)						
< 50	42 (15.7)	226 (84.3)	1	< 0.001	1	< 0.001
≥ 50	36 (42.4)	49 (57.6)	3.95 (2.30–6.80)		3.85 (2.03–7.31)	
Sex						
Male	15 (10.3)	130 (89.7)	1	< 0.001	1	< 0.001
Female	63 (30.3)	145 (69.7)	3.77 (2.04–6.94)		4.18 (2.08–8.40)	
Residence						
Urban	56 (24.7)	171 (75.3)	1.55 (0.89–2.68)	0.118	1.06 (0.56–2.03)	0.858
Rural	22 (17.5)	104 (82.5)	1		1	
Education						
< High school	65 (22.3)	226 (77.7)	1.08 (0.55–2.12)	0.814		
≥ High school	13 (21.0)	49 (79.0)	1			
Baseline CD4 count (cells/mm3)						
< 200	31 (34.4)	59 (65.6)	2.42 (1.41–4.13)	0.001	2.41 (1.24–4.69)	0.009
≥ 200	47 (17.9)	216 (82.1)	1		1	
Current CD4 count (cells/mm3)						
< 200	27 (40.3)	40 (59.7)	3.11 (1.75–5.52)	< 0.001	2.32 (1.15–4.68)	0.018
≥ 200	51 (17.8)	235 (82.2)	1		1	
WHO Clinical Stage						
1/2	67 (22.1)	236 (77.9)	1.01 (0.49–2.07)	0.986		
3/4	11 (22.0)	39 (78.0)	1			
Body mass index (Kg/m2)						

< 25	49 (17.3)	234 (82.7)	1	< 0.001	1	0.002
≥ 25	29 (41.4)	41 (58.6)	3.38 (1.92–5.95)		2.91 (1.49–5.71)	
Duration on ART (years)						
≤ 3	31 (17.4)	147 (82.6)	1	0.033	1	0.269
> 3	47 (26.9)	128 (73.1)	1.74 (1.04–2.90)		0.66 (0.32–1.37)	
ART Regimen						
TDF-based	27 (20.5)	105 (79.5)	0.86 (0.51–1.45)	0.566		
Non-TDF based	51 (23.1)	170 (76.9)	1			
Hemoglobin (g/dl)						
< 12	30 (42.9)	40 (57.1)	3.67 (2.09–6.47)	< 0.001	3.38 (2.00–7.46)	0.001
≥ 12	48 (17.0)	235 (83.0)	1		1	
Baseline eGFR (ml/min/1.73m^2)						
< 60	28 (40.0)	42 (60.0)	3.11 (1.76–5.48)	< 0.001	1.10 (0.54–2.24)	0.596
≥ 60	50 (17.7)	233 (82.3)	1		1	

ART, antiretroviral therapy; eGFR, estimated glomerular filtration rate; TDF, tenofovir

Discussion

In this study, we evaluated renal function among HIV-positive individuals at baseline prior to ART initiation and during follow-up. At baseline before ART initiation, 48.8% of the patients had eGFR < 90 ml/min/1.73 m^2. Of these 19.8% had eGFR < 60 ml/min/1.73 m^2 and 28.9% had eGFR 60–89.9 ml/min/1.73 m^2. Factors associated with baseline renal impairment (eGFR <60 ml/min/1.73m m^2) were female sex, lower CD4 count, lower BMI, lower hemoglobin and higher total cholesterol. Our findings are comparable to some of the studies in the region regarding the prevalence of renal impairment at baseline prior to ART initiation using the same definition with MDRD formula (21,27). Other studies showed highly variable prevalence according to the racial distribution, clinical characteristics, and immunological control. In a large cohorts of HIV-positive subjects, studies in Burkina Faso (28), China (29), Spain (30) and Nigeria (19) reported prevalence of renal impairment (eGFR < 60 ml/min/1.73 m^2) of 3.0%, 3.3%, 5% and 24%, respectively at baseline before ART initiation using the same MDRD formula for eGFR. These findings were concerning given reports that impaired renal function at baseline is an independent predictor of death and renal disease progression in HIV-infected patients (4,5).

Our study documented risk factors for renal impairment (eGFR < 60 ml/min/1.73 m^2) in patients with HIV at the baseline prior to ART initiation that are similar to those identified by previous

studies including female sex, low CD4 count, low BMI, and low hemoglobin (21,29,31–34). In this study, older age at baseline was also significantly associated with increased risk of renal impairment before initiating ART in univariate analyses, but did not obtain statistical significance in multivariate analysis. Multiple previous studies in cohorts of HIV-positive subjects initiating ART reported older age as a strong risk factor of impaired renal function at treatment initiation (21,29,30,32–34). Interestingly in our study we found higher total cholesterol associated with baseline renal impairment in patients with HIV. The underlying pathophysiologic mechanisms for the relationship between lipid levels and loss of renal function are not yet fully understood, although there are data supporting a causal relationship between baseline lipids and the development of renal impairment (35).

About 22.1% of patients on ART in our study had renal impairment (eGFR < 60 ml/min/1.73 m^2). This finding is higher than that reported from a study conducted in Northwest Ethiopia where renal impairment was found in 11.7% of the HIV-infected patients on ART (22), and from studies in Tanzania (1.2%) (21), and Ghana (5.2% and 9.9%) (36,37) who used the same MDRD formula. The differences could be due to variation in the severity of HIV infection, types and duration of ART, or due to variation in the lifestyle and age distribution of study patients. Analysis of the present study showed a significant increase in the prevalence of renal impairment after a median ART duration of 3.0 years, from 19.8% at baseline to 22.1%. This was consistent with findings reported from the Ethiopian study; which showed an increase in prevalence of renal impairment from 3.6% before to 11.7% after ART (22). In contrast, the above sub-Saharan African cohort studies reported a significant decrease in the prevalence of renal impairment after initiating ART (20,21). These studies however, reported less renal impairment following ART initiation among individuals starting therapy with advanced disease, median CD4 cell counts < 143 cells/mm^2. The disparity can also be explained by differences in the etiologies of renal impairment. For example, HIV-related renal impairment, which responds to ART, is absent among Ethiopian descents (38,39) and ART is not always beneficial in improving renal function in this context (10,18).

The present study also revealed a decline in mean eGFR after ART initiation, from a mean eGFR of 112.9 ± 81.2 ml/min/1.73 m^2 at baseline to 93.9 ± 60.6 ml/min/1.73 m^2 after a median ART follow-up of 3.0 years. A study in Ghana similarly showed a progressive decline in mean eGFR over the period whilst on ART (36). In the ICONA Foundation cohort study, Tordato et al. (34)

observed a decrease in eGFR of at least 20% from pre-ART levels in patients on ART who were drug-naıve at baseline. In the Asian study by Suzuki et al (40), a faster rate of eGFR decline was also observed after ART initiation, with a median duration of 3.07 years. The decline in eGFR emerges in patients on ART for no less than 12 weeks after the start of combination ART, and then tended to decrease gradually thereafter (41). However, an improvement in eGFR after the commencement of ART has been demonstrated in the Tanzanian, South African and Ugandaian cohort studies (20,21,31). Our results also showed that among patients with baseline normal renal function (eGFR ≥90 ml/min/1.73 m^2), 49.7% experienced some degree of renal impairment, which is similar to recent studies done in Tanzania and China (29,32). Likewise, in the analysis of patients who initiated ART with normal renal function a significant proportion of patients have been shown to experience a decrease in eGFR to a value of < 60 or < 90 mL/min/1.73 m^2 after ART initiation (34,42). These findings have particular importance in our settings, where monitoring of renal function is often insufficient due to limited resources.

Older age, being female, low current CD4 count, and low hemoglobin were associated with renal impairment among HIV-infected patients on ART. These findings are similar to those of other related studies (29,31,32,34,36,42,43). Older patients are at increased risk for renal impairment as a result of increasing comorbidity and a physiologic decline in GFR with age (13). Aging and gender are a non-modifiable risk factors that played a significant role in the development of renal impairment in our patients during follow-up, suggesting the need to implement possible preventive measures in these sub-group of population for reducing progression to renal failure and other adverse events. On the other hand, maintenance of high CD4 counts and of normal hemoglobin levels may be potentially modifiable factors that can reduce the risk of renal impairment in HIV-infected patients on ART. Similar to the South African study, a high BMI has been identified as a risk factor for renal impairment in our patients after ART initiation (31).

Lower baseline CD4 count has also been identified as risk factors for renal impairment after ART initiation. This is consistent with findings from related studies, which reported that lower baseline CD4 count may have an additive effect on renal impairment risk in HIV infected patients after ART initiation (21,29,40,43). We found no significant differences in renal impairment among patients on TDF and non–TDF containing ART regimens. Similar findings were observed in an African studies (21,31,44). The Myanmar study by Kyaw et al examining nearly 1400 HIV

infected patients starting first line TDF based ART has also shown a low incidence of renal impairment and suggested that routine assessment of renal function may not be needed among those on a TDF-containing regimens (43). These findings are reassuring for clinicians in low income settings where TDF is massively prescribed as part of first-line ART regimen. However, statistically significant reductions in renal function after the initiation of TDF, compared with after the initiation of other drugs, have been reported in other studies (34,40,41). In this regard, results from a systematic review identified studies in Africans reporting statistically significant renal function decline associated with TDF use (45). In the Ethiopian cohort study by Yazie et al (46), an eGFR decrease of >25% from baseline was observed in 25.4% of patients initiating TDF containing combination ART. Future prospective study is therefore required with large sample size and long duration to ascertain the association of TDF with renal impairment among Ethiopian patients initiating TDF-based ART regimens.

Limitations

First, this is an observational cohort study and not a randomized clinical trial. Second, some clinical indicators of renal health or treatment (e.g. proteinuria and viral loads) were not available in our database and are not included in this cohort. Third, although subjects in this analysis were exposed to multiple ART regimens, the effects of specific ART regimens on renal function was not assessed. Fourth, the MDRD study equation has not been modified for use in HIV-positive Ethiopian patients. Lastly, the measurement of serum creatinine was not standardized; this might influence the performance of eGFR equation. This also hindered us from using the popular Chronic Kidney Disease Epidemiology Collaboration equation.

Conclusions

In conclusion, impaired renal function was common in HIV-infected patients initiating ART in an outpatient setting in Ethiopia, and there appears to be a high prevalence of renal impairment after a median ART follow-up of 3 years. Our data highlight the need for assessment of renal function at baseline before ART initiation and regular monitoring of renal function for patients with HIV during follow-up, with aggressive management of risk factors. Further well-controlled cohort

studies for the evaluation of long-term effects of ART on renal function are needed to confirm our findings.

Abbreviations

AOR: Adjusted odds ratio; ART, antiretroviral therapy; BMI: body mass index; CI: Confidence interval; eGFR: estimated glomerular filtration rate; MDRD: Modification of Diet in Renal Disease; OR: odds ratio.

Acknowledgements

The authors would like to acknowledge staffs of ART clinic of Mehal Meda Hospital for collecting the data

References

1. Szczech LA, Hoover DR, Feldman JG, Cohen MH, Gange SJ, Gooze L, et al. Association between Renal Disease and Outcomes among HIV-Infected Women Receiving or Not Receiving Antiretroviral Therapy. CID. 2004;39:1199–206.

2. Choi AI, Li Y, Deeks SG, Grunfeld C, Volberding PA, Shlipak MG. Association Between Kidney Function and Albuminuria With Cardiovascular Events in HIV-Infected Persons. Circulation. 2010;121:651–8.

3. Mocroft A, Ryom L, Begovac J, Monforte AD, Vassilenko A, Gatell J, et al. Deteriorating renal function and clinical outcomes in HIV-positive persons. AIDS. 2014;28:727–37.

4. Estrella MM, Parekh RS, Abraham A, Astor BC, Szczech LA, Anastos K, et al. The Impact of Kidney Function at Highly Active Antiretroviral Therapy Initiation on Mortality in HIV-Infected Women. J Acquir Immune Defic Syndr. 2010;55:217–20.

5. Ibrahim F, Hamzah L, Jones R, Nitsch D, Sabin C, Post FA, et al. Baseline Kidney Function as Predictor of Mortality and Kidney Disease Progression in HIV-Positive Patients. Am J Kidney Dis. 2012;60(4):539–47.

6. Gardner LI, Klein RS, Szczech LA, Phelps RM, Tashima K, Rompalo AM, et al. Rates and Risk Factors for Condition-Specific Hospitalizations in HIV-infected and Uninfected Women. J Acquir Immune Defic Syndr. 2003;34(3):320–30.

7. Ibrahim F, Naftalin C, Cheserem E, Roe J, Campbell LJ, Bansi L, et al. Immunodeficiency and renal impairment are risk factors for HIV-associated acute renal failure. AIDS. 2010;24:2239–44.

8. Wearne N, Davidson B, Blockman M, Swart A, Jones ESW. HIV, drugs and the kidney. Drugs Context. 9: 2019-11–1.

9. Gupta S, Eustace J, Winston J. Guidelines for the management of chronic kidney disease in HIV-infected patientes: recomendations of the HIV Medicine Association of the infectious diseases society of America. Clin Infect Dis. 2005;40:1559–85.

10. Szczech LA, Gupta SK, Habash R, Guasch A, Kalayjian R, Appel R, et al. The clinical epidemiology and course of the spectrum of renal diseases associated with HIV infection. Kidney Int. 2004;66:1145–52.

11. Roling J, Schmid H, Fischereder M, Draenert R, Goebel F. HIV-Associated Renal Diseases and Highly Active Antiretroviral Therapy–Induced Nephropathy. CID. 2006;42:1488–95.

12. Atta MG, Gallant JE, Rahman MH, Nagajothi N, Racusen LC, Scheel PJ, et al. Antiretroviral therapy in the treatment of HIV-associated nephropathy. Nephrol Dial Transpl. 2006;21:2809–13.

13. Nadkarni GN, Konstantinidis I, Wyatt CM. HIV and the aging kidney. Curr Opin HIV AIDS. 2014;9:340–5.

14. Jung O, Haack HS, Brodt H-R, Grützmacher P, Geiger H, Amann K, et al. Changing Spectrum of Renal Disease in HIV Infection. Dtsch Med Wochenschr. 2013;138(38):1887–91.

15. Mocroft A, Lundgren JD, Ross M, Fux CA, Reiss P, Moranne O, et al. Cumulative and current exposure to potentially nephrotoxic antiretrovirals and development of chronic kidney disease in HIV-positive individuals with a normal baseline estimated glomerular filtration rate: a prospective international cohort study. Lancet HIV. 2016;3:e23–32.

16. Ryom L, Mocroft A, Kirk O, Worm SW, Kamara DA, Reiss P, et al. Association Between Antiretroviral Exposure and Renal Impairment Among HIV-Positive Persons With Normal Baseline Renal Function: the D:A:D Study. JID. 2013;207:1359–69.

17. Hou J, Nast CC. Changing concepts of HIV infection and renal disease. Curr Opin Nephrol Hypertens. 2018;27(3):144–52.

18. Ding Y, Duan S, Ye R, Yao S, Cao D, Yang Y, et al. Effects of aging, baseline renal function and stage of HIV infection on post-treatment changes in renal function among HIV-infected patients: a retrospective cohort study. HIV Med. 2019;20(9):591–600.

19. Adedeji TA, Adedeji NO, Adebisi SA, Idowu AA, Fawale MB, Jimoh KA. Prevalence and Pattern of Chronic Kidney Disease in Antiretroviral-Naı ¨ve Patients with HIV/AIDS. J Int Assoc Provid AIDS Care. 2015;14(5):434–40.

20. Peters PJ, Moore DM, Mermin J, Brooks JT, Downing R, Were W, et al. Antiretroviral therapy improves renal function among HIV-infected Ugandans. Kidney Int. 2008;74:925–9.

21. Mpondo BCT, Kalluvya SE, Peck RN, Kabangila R, Kidenya BR, Ephraim L, et al. Impact of Antiretroviral Therapy on Renal Function among HIV-Infected Tanzanian Adults: A Retrospective Cohort Study. PLoS ONE. 2014;9(2):e89573.

22. Baynes HW, Tegene B, Gebremichael M, Birhane G, Kedir W, Biadgo B. Assessment of the effect of antiretroviral therapy on renal and liver functions among HIV-infected patients: a retrospective study. HIV/AIDS. 2017;9:1–7.

23. Kooij KW, Vogt L, Wit FWNM, Valk M van der, Zoest RA van, Goorhuis A, et al. Higher Prevalence and Faster Progression of Chronic Kidney Disease in Human Immunodeficiency Virus– Infected Middle-Aged Individuals Compared With Human Immunodeficiency Virus–Uninfected Controls. JID. 2017;216:622–31.

24. Choi AI, Shlipak MG, Hunt PW, Martin JN, Deeks SG. HIV-infected persons continue to lose kidney function despite successful antiretroviral therapy. AIDS. 2009;23(16):2143–9.

25. Levey A, Greene T, Kusek J, Beck G. A simplified equation to predict glomerular filtration rate from serum creatinine. J Am Soc Nephrol. 2000;11:A0828.

26. American Kidney Foundation. Clinical practice guidelines for chronic kidney disease: evaluation, classification, and stratification. Am J Kidney Dis 2002; 39 (2 Suppl 1): S1–S266.

27. Msango L, Downs JA, Kalluvya SE, Kidenya BR, Kabangila R, Johnson Jr. WD, et al. Renal Dysfunction among HIV-Infected Patients Starting Antiretroviral Therapy in Mwanza, Tanzania. AIDS. 2011;25(11):1421–5.

28. Kaboré NF, Poda A, Zoungrana J, Da O, Ciaffi L, Semdé A, et al. Chronic kidney disease and HIV in the era of antiretroviral treatment: findings from a 10-year cohort study in a west African setting. BMC Nephrol. 2019;20:155.

29. Zhao Y, Zhang M, Shi CX, Zhang Y, Cai W, Zhao Q, et al. Renal Function in Chinese HIV-Positive Individuals following Initiation of Antiretroviral Therapy. PLoS ONE. 2015;10(8):e0135462.

30. Juega-Mariño J, Bonjoch A, Pérez-Alvarez N, Negredo E, Bayes B, Bonet J, et al. Prevalence, evolution, and related risk factors of kidney disease among Spanish HIV-infected individuals. Medicine (Baltimore). 2017;96:37(e7421).

31. Assaram S, Mashamba-Thompson TP, Magula NP. Risk factors and co-morbidities associated with changes in renal function among antiretroviral treatment-naïve adults in South Africa: A chart review. Afr J HIV Med. 2018;19(1):a770.

32. Mapesi H, Kalinjuma AV, Ngerecha A, Franzeck F, Hatz C, Tanner M, et al. Prevalence and Evolution of Renal Impairment in People Living With HIV in Rural Tanzania. Open Forum Infect Dis. 2018;5(4):ofy072.

33. Mocroft A, Kirk O, Gatell J, Reiss P, Gargalianos P, Zilmer K, et al. Chronic renal failure among HIV-1-infected patients. AIDS. 2007;21:1119–27.

34. Tordato F, Cozzi Lepri A, Cicconi P, De Luca A, Antinori A, Colangeli V, et al. Evaluation of glomerular filtration rate in HIV-1-infected patients before and after combined antiretroviral therapy exposure. HIV Med. 2014;12:4–13.

35. Schaeffner ES, Kurth T, Curhan GC, Glynn RJ, Rexrode KM, Baigent C, et al. Cholesterol and the risk of renal dysfunction in apparently healthy men. J Am Soc Nephrol. 2003;14(8):2084–91.

36. Obiri-Yeboah D, Awuku YA, Alofa W, Charwudzi A, Aniakwa-Bonsu E, Obboh E, et al. Renal dysfunction among adult HIV/AIDS patients on antiretroviral therapy at a tertiary facility in Ghana. BMC Nephrol. 2018;19:333.

37. Obirikorang C, Osakunor DNM, Ntaadu B, Adarkwa OK. Renal Function in Ghanaian HIV-Infected Patients on Highly Active Antiretroviral Therapy: A Case-Control Study. PLoS ONE. 2014;9(6):e99469.

38. Behar DM, Shlush LI, Maor C, Lorber M, Skorecki K. Absence of HIV-Associated Nephropathy in Ethiopians. Am J Kidney Dis. 47:88–94.

39. Behar DM, Kedem E, Rosset S, Haileselassie Y, Tzur S, Kra-Oz Z, et al. Absence of APOL1 Risk Variants Protects against HIV-Associated Nephropathy in the Ethiopian Population. Am J Nephrol. 2011;34:452–9.

40. Suzuki S, Nishijima T, Kawasaki Y, Kurosawa T, Mutoh Y, Kikuchi Y, et al. Effect of Tenofovir Disoproxil Fumarate on Incidence of Chronic Kidney Disease and Rate of Estimated Glomerular Filtration Rate Decrement in HIV-1–Infected Treatment-Naı ̈ve Asian Patients: Results from 12-Year Observational Cohort. AIDS PATIENT CARE STDs. 2017;31(3):105–12.

41. Tanaka H, Mariko Arai, Tomoda Y, Wada T, Yago K, Satoh M. Evaluation of Renal Adverse Effects of Combination Anti-retroviral Therapy including Tenofovir in HIV-infected Patients. J Pharm Pharm Sci. 2013;16(3):405–13.

42. Domingo P, Suarez-Lozano I, Gutierrez F, Estrada V, Knobel H, Palacios R, et al. Predictive factors of renal impairment in HIV-infected patients on antiretroviral therapy: Results from the VACH longitudinal cohort study. Nefrologia. 2019;39(5):497–505.

43. Kyaw NTT, Harries AD, Chinnakali P, Antierens A, Soe KP, Woodman M, et al. Low Incidence of Renal Dysfunction among HIV-Infected Patients on a Tenofovir-Based First Line Antiretroviral Treatment Regimen in Myanmar. PLoS ONE. 2015;10(8):e0135188.

44. Salome T, Kasamba I, Mayanja BN, Kazooba P, Were J, Kaleebu P, et al. The effect of Tenofovir on renal function among Ugandan adults on long-term antiretroviral therapy: a cross-sectional enrolment analysis. AIDS Res Ther. 2016;13:28.

45. Mtisi TJ, Ndhlovu CE, Maponga CC, Morse GD. Tenofovir-associated kidney disease in Africans: a systematic review. AIDS Res Ther. 2019;16:12.

46. Yazie TS, Orjino TA, Degu WA. Reduced Kidney Function in Tenofovir Disoproxil Fumarate Based Regimen and Associated Factors: A Hospital Based Prospective Observational Study in Ethiopian Patients. Int J Nephrol. 2019;2019:1–9.

Figure

Click here to download Figure Capture eGFR.jpg

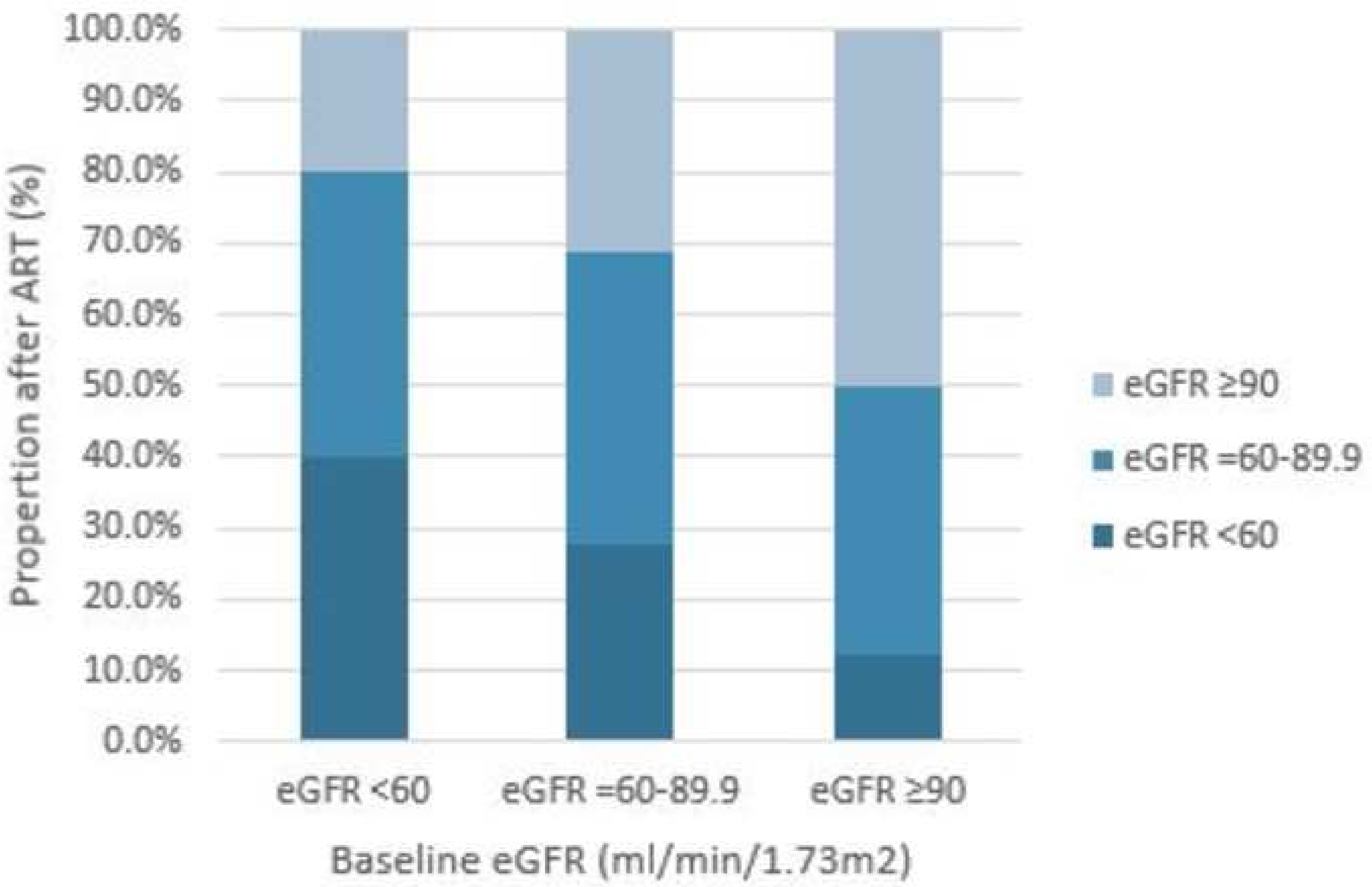

Supplement: Attachment — Submitted filename: PONE-D-20-19005_reviewer.pdf [file pone.0245500.s004.pdf]
