# Supplementary material for: Renal function in a cohort of HIV-infected patients initiating antiretroviral therapy in an outpatient setting in Ethiopia
Source: PLoS One. 2021 Jan 22;16(1):e0245500. doi: 10.1371/journal.pone.0245500 (PMC7822244; doi:10.1371/journal.pone.0245500)
Supplement: Attachment — Submitted filename: Review commentsPLOSone20200814.docx [file pone.0245500.s003.docx]

**Review comments**

Important study looking at the burden of Renal impairment among HIV infected adults in the ART era. However, there are issues that must be addressed first before first interpreting the findings and then considering this paper for publication.

**Major Issues**

1. First, patients with missing data were dropped and only those who had data were analyzed. If were are to infer the findings presented here we need to know that they represent the underlying population (denominator) and hence they are from a random sample of the target population. Until details are provided this data as it is suggests a high possibility of non-random selection of participants making the study prone to selection bias. To evaluate this study, we need to know number, proportion and characteristics of patients who were dropped. Usually a flow diagram will be useful to demonstrate this.
2. Secondly, the analytical approach used is incongruent with the study design. While a cohort design was used only the prevalence analyses and logistic regression where used. Actually for the baseline prevalence assessment was okay however upon follow-up a longitudinal analysis that accounts for time in follow-up should be used both for disease burden and multivariate regression. This means a time-to-event analysis and a pooled logistic regression or poisson regression or proportional hazards regression etc… should be considered. This warrants a reanalysis if this paper is to be considered for publication.
3. Since the outcome, renal impairment, could be precluded by death as a competing risk, this also will have to be considered as well with the appropriate survival analysis technique for the descriptive analysis and hence consider the Aalen-Johansen estimator.
4. Characteristics of the cohort are not described well. While time zero is stated as ART initiation, end of follow-up, transfer out, loss to follow up, death as a competing risk, database closure etc… are all not defined nor described. Also the respective numbers and proportions of how these outcomes affected the study population during follow-up are not provided. This must be addressed if we are to interpret the findings appropriately.
5. In this HIV infected population on ART, regressions didn’t consider any HIV related immological and virological parameters. Opportunistic infections that could impair the kidneys either directly or due to their treatments e.g. CCM and sepsis from any cause were also not accounted for either as mediators or confounders.
6. Another aspect that might need to be considered for the analysis is the within and between subject variation overtime for the renal impairment. Risk of renal impairment depends of the individual and varies with time depending on what the patients is exposed to.
