# Supplementary material for: Renal function in a cohort of HIV-infected patients initiating antiretroviral therapy in an outpatient setting in Ethiopia
Source: PLoS One. 2021 Jan 22;16(1):e0245500. doi: 10.1371/journal.pone.0245500 (PMC7822244; doi:10.1371/journal.pone.0245500)
Supplement: Attachment — Submitted filename: Response to reviewer- PONE-2020.doc [file pone.0245500.s005.doc]

Cover letter

Temesgen Fiseha

Wollo University

Dessie, Ethiopia

Email: [temafiseha@gmail.com](mailto:temafiseha@gmail.com)

**Date:** October 20, 2020

**To: PLOS ONE Journal editorial office**

**Dear Editorial:**

We are glad to write this response that our paper entitled **“Renal function in a cohort of HIV-infected patients initiating antiretroviral therapy in an outpatient setting in Ethiopia”** has been considered for publication in PLOS ONE. We are pleased to have an opportunity to make our paper revised and we have greatly appreciated the reviewers’ comments, and suggestions were very helpful overall. In revising the paper, we have carefully considered reviewers’ comments and suggestions on our revised submission. As instructed, we have attempted to succinctly explain changes made in reaction to all comments and reply to each comment in point-by-point fashion as follows:

**Response to Reviewer Comments**

**Reviewer #1**

**Comment # 1:** *First, patients with missing data were dropped and only those who had data were analyzed. If were are to infer the findings presented here we need to know that they represent the underlying population (denominator) and hence they are from a random sample of the target population. Until details are provided this data as it is suggests a high possibility of non-random selection of participants making the study prone to selection bias. To evaluate this study, we need to know number, proportion and characteristics of patients who were dropped. Usually a flow diagram will be useful to demonstrate this.*

**Response** #**1**: As suggested, it is stated in the result section as “Six hundred and forty-eight adults ≥ 18 years old were followed in this outpatient ART clinic for at least six months. A total of 295 were excluded: 143 did not have recorded baseline creatinine result before ART initiation, 40 did not have creatinine results after initiating ART at the most recent visit, and 112 patients had missing data or lost to follow up (Fig. [1](https://bmcinfectdis.biomedcentral.com/articles/10.1186/s12879-020-05308-9#Fig1)). The excluded patients were similar to those who were included in this analysis in terms of age (mean 37.3 ± 10.0 years) and gender (60.0% female) distributions. The mean baseline CD4 count was 398.6 ± 217 cells/mm^3^ in the excluded HIV-infected patients and 50 (16.9%) were in WHO clinical stage 3/4.” (Line 153-159)

Fig 1. Flow diagram showing the number of patients included in the analysis

**Comment # 2:** *Secondly, the analytical approach used is incongruent with the study design. While a cohort design was used only the prevalence analyses and logistic regression where used. Actually for the baseline prevalence assessment was okay however upon follow-up a longitudinal analysis that accounts for time in follow-up should be used both for disease burden and multivariate regression. This means a time-to-event analysis and a pooled logistic regression or poisson regression or proportional hazards regression etc… should be considered. This warrants a reanalysis if this paper is to be considered for publication.*

**Response** #**2**: As stated in the introduction section the objective of our study was to evaluate the prevalence and associated risk factors of abnormal renal function before ART initiation (at baseline) and after ART initiation (after a median follow-up time of 3 years of ART treatment) and to describe the impact of ART on renal function in HIV-infected patients initiating ART using data collected before and after the ART treatment. (Line 102-109). Also as stated in the method section “After initiation of therapy, if a patient had multiple documented serum creatinine results, only the most recent result was utilized for the analysis” (Line 102-109), serum creatinine readings at the most recent measurement (i.e., at the most recent follow-up visit) was used for renal function assessment after ART treatment (during ART follow-up). As such statistical analysis of one group pre- and post-treatment comparison with one post-treatment measurement was employed, and it was stated in the method statistical analysis section as “Paired t-test was used to evaluate mean eGFR and CD4 T cell count difference before and after ART initiation. McNemar’s test was used to compare the proportion of patients with renal impairment before and after ART initiation.” (Line 144-146)

**Comment # 3:** *Since the outcome, renal impairment, could be precluded by death as a competing risk, this also will have to be considered as well with the appropriate survival analysis technique for the descriptive analysis and hence consider the Aalen-Johansen estimator.*

**Response** #**3**: As stated in response # 2, our objective was to evaluate the prevalence and associated risk factors of abnormal renal function before (at baseline) and during ART and to describe the impact of ART on renal function. HIV-positive patients are observed/measured twice and we compared values of outcome variable before and after ART treatment. Univariate and multivariate regression analysis were used to determine factors associated with renal impairment at the baseline prior to and after ART treatment. The present study does not focus on survival analysis/longitudinal analysis, because data on death at each time point; i.e., ≥ 3 time-to-event occurrence are needed (which is beyond our objective).

**Comment # 4:** *Characteristics of the cohort are not described well. While time zero is stated as ART initiation, end of follow-up, transfer out, loss to follow up, death as a competing risk, database closure etc… are all not defined nor described. Also the respective numbers and proportions of how these outcomes affected the study population during follow-up are not provided. This must be addressed if we are to interpret the findings appropriately.*

**Response** #**4**: Characteristics of the subjects described in the manuscript and in Table 1, and the proportion of patients excluded from the study are also described, as stated in response # 2. We collected repeated measures data where subjects are only measured twice, i.e., we have only one group of subjects and have measured them on two occasions only, before (at the baseline) and after ART initiating (at their last visit). As such the respective numbers and proportions of how these outcomes affected the study population during follow-up are not provided, a situation where more than two (≥ 3) measurements per subject are needed.

**Comment # 5:** *In this HIV infected population on ART, regressions didn’t consider any HIV related immological and virological parameters. Opportunistic infections that could impair the kidneys either directly or due to their treatments e.g. CCM and sepsis from any cause were also not accounted for either as mediators or confounders.*

**Response** #**5**: HIV related immological parameter (CD4+ T cell count) was considered in the study analysis both before and after ART initiation and as stated in the result section (Line 179-180), paired t-test was used to assess mean differences for CD4+ T cell count before and after ART initiation. Being resource limited setting, measurements of virological parameters (viral load) are rarely performed in our setting and thus are not considered in the study analysis. Opportunistic infections that could impair the kidneys either directly or due to their treatments e.g. CCM and sepsis from any cause were also not accounted for either as mediators or confounders because such data are missing for most of the study patients, especially during ART at their last visit.

**Comment # 6:** *Another aspect that might need to be considered for the analysis is the within and between subject variation overtime for the renal impairment. Risk of renal impairment depends of the individual and varies with time depending on what the patients is exposed to.*

**Response** #**6**: In the present study repeated measurements are taken from one group of participants on two occasions only, before (at baseline) and after ART treatment (at their last visits), differences in mean eGFR or differences in the proportion of patients with renal impairment before and after ART was compared using a [paired sample test](https://help.xlstat.com/s/article/students-t-test-on-two-paired-samples-tutorial?language=en_US) (within-subjects) as stated in response # 2. Compressions between groups both at the baseline and on ART were made using Chi-square test for categorical variables and student’s t-test for continues variables, as stated in the method statistical analysis section (Line 1141-142). As such between subject variation overtime (between-subject analysis) to compare renal function for two groups of participant (i.e., treatment and control groups) was not performed. Also analysis of repeated measures taken overtime (involving ≥3 measurements) were not performed using a linear regression models approach to assess the effect of time-dependent covariates on eGFR or to exhibit correlation within an individual overtime.

**Reviewer #2**

The authors looked at the renal function in a cohort of HIV patients in Ethiopia. The manuscript is well written and the language is acceptable. My comments are attached as sticky pads in the attached PDF file.

**Comment # 1:** *How were these patients factored in the data analysis since diabetes and hypertension are confounding factors for CKD?*

**Response** #**1**: Diabetes and hypertensive patients had increased odds of renal impairment at baseline (Mocroft et al. 2007). These patients are factored in the data analysis to investigate these comorbid renal risk factors are associated with increased risk of prevalent renal impairment (non-HIV associated renal impairment), because they were thought to present significant challenges to HIV management especially in resource limited settings like our setting. Strategies that use baseline comorbidities, such as diabetes and hypertension, to identify individuals at high risk of renal impairment at the time of ART initiation may be effective.

**Comment # 2:** *Aside comparing your study with other similar/different studies, what is accounting for the results obtained. What are the reasons/pathophysiology for the results obtained?*

**Response** #**2**: As suggested, it is stated as “The disparity seems to be explained mainly by the characteristics of studied patients or by the differences in the creatinine assays and calibration. It can thus be inferred that HIV as a disease state is associated with renal function impairment independent of the possible nephrotoxic effects of antiretroviral agents used in the treatment of persons with HIV. The basis of HIV-induced renal impairment has been recognized as direct cellular injury by the virus or by changes in the release of cytokines during HIV infection (19,31).” (Line 239-244)

**Comment # 3:** *There is a fine mechanism that explains the relationship between dyslipidamia (high cholesterol) and renal impairment.*

**Response** #**3**: As suggested, it is stated as “The underlying pathophysiologic mechanisms for the relationship between lipid levels and loss of renal function are not yet fully understood, although there are data that oxidative stress and insulin resistance may mediate the lipid-induced renal impairment (36). (Line 253-256)

**Comment # 4:** *What are the reasons accounting for the increase or decrease in the in eGFR in patients on ART?*

**Response** #**4**: As suggested, it is stated as “Exposure to HIV viremia, chronic inflammation, and potentially nephrotoxic antiretroviral drugs, as well as structural or functional disorders in the context of ageing and associated comorbidities may play a role in the pathogenesis of eGFR decline in HIV-infected patients on ART (23,43).” (Line 281-284)

**Comment # 5:** *Age is a confounding factor for a decline in eGFR. How different is that in HIV patients?*

**Response** #**5**: It is different in HIV-infected patients, particularly in patients exposed to ART. As suggested, it is stated as “Older patients are at increased risk for renal impairment as a result of increasing comorbid risk factors such as diabetes and hypertension, which are prevalent in HIV-infected patients on ART, or due to a physiologic decline in GFR with age (13).” (Line 295-297)

**Comment # 6:** *What accounts for a high BMI being a risk factor for renal impairment?*

**Response** #**6**: As suggested, it is stated as “ High BMI after ART-initiation is associated with cardiometabolic complications including atherothrombotic cardiovascular disease and diabetes that increase the risk for renal impairment during HIV treatment (46,47).” (Line 304-307)

**Comment # 7:** *Reasons for these differences in 'similar' populations should have been stated*.

**Response** #**6**: As suggested, it is stated as “Differences in study design, definition of renal impairment and duration of TDF exposure may partially explain the discrepancy between the results of the present study and the previous study in the country.” (Line 323-325)

**Reviewer #3**

This is a pertinent topic which the authors can improve.

**Comment # 1:** *The introduction would benefit from a better detailed literature review that puts into better context the problem statement for Ethiopians. A more robust literature review is needed to strengthen the article.*

**Response** #**1**: As suggested, it is stated as “Despite the introduction of the ‘test and treat’ program in Ethiopia, little is known about the status of renal function in HIV-infected individuals at the time of initiation of ART and during ART in this resource-limited settings. Renal function pre-ART and during ART is not routinely performed. The aim of … “(Line 105-108)

**Comment # 2:** *The study method, while it is a retrospective cohort, should be better stated as Retrospective "observational" cohort as this describes upfront the specific cohort methodology employed.*

**Response** #**2**: As suggested, it is stated as “Retrospective observational cohort study” (Line 32, 114)

**Comment # 3:** *In line 114, authors state, "The inclusion criteria were age older than 18 years..." This implies that patients who were 18 years were excluded. The statement should be re-written to "...were 18 years and older" as it is clear they included 18 year olds in the study*

**Response** #**3**: As suggested, it is stated as "...were 18 years and older" (Line 117)

**Comment # 4:** *Lines 117-118. "The study protocol was approved by the Institutional Review Board of College of Medicine and Health Sciences, Wollo University." Authors must provide the approval number for the study.*

**Response** #**4**: As suggested, it is stated as "...*, Wollo University* (# 133/13/12).” (Line 122)

**Comment # 5:** *The authors declare that this study did not obtain ethical approval. This is a study that involves human participants and therefore requires ethical approval as it involves access to and use of medical records. If approval was not obtained, authors must explain why. in the very least, a waiver of ethical approval letter must be obtained from the ethics review committee.*

**Response** #**5**: As suggested, it is stated as “Written informed consent was not given by participants for their clinical records to be used in this study, but patient’s identifiers were removed and only code numbers were used throughout the study.” (Line 122-124)

**Comment # 6:** *The authors declare that all data is available in a public domain. Is this with reference to the primary medical records used for the study? If this is the case, ethics approval/consent from the patients or waiver thereof must be in place to provide for this.*

**Response** #**6:** This is not with reference to the primary medical records, but for the database used in this manuscript. As stated in response # 5; To ensure confidentiality, patient’s identifiers were removed and only code numbers were used throughout the study.

**Comment # 7:** *May the authors also clarify where (the repository) the data in comment 6 is available?*

**Response** #**7:** The data used in the study manuscript will be available as a supplementary file. As suggested, it is stated as “The database for this manuscript is available as an additional supporting information (S1 Dataset).” (Line 124-125) Supporting Information. S1 Dataset. The Excel database used for this manuscript. (XLS) (Line 347-349)

**Comment # 8:** *Limitations; In stating the limitations to the study, line 306, outline how the limitation impacted the results.*

**Response** #**8:** This is to mean that the study lacked a control group or participants are not randomized to 2 arms (a randomized control-group pre- and post- treatment study is required to investigate the effects of ART on renal function).

**Comment # 9:** *Minor correction: Please correct the typographical error on Reference number 19, line 384. The spelling of Naive was captured incorrectly in the reference manager*

**Response** #**9:** As suggested, it is *spelled* as "Naive” (Line 407)

Looking forward to hearing from you. Thank you again for your consideration!

Sincerely,

Temesgen Fiseha (BSc, MSc)
