# Supplementary material for: Renal function in a cohort of HIV-infected patients initiating antiretroviral therapy in an outpatient setting in Ethiopia
Source: PLoS One. 2021 Jan 22;16(1):e0245500. doi: 10.1371/journal.pone.0245500 (PMC7822244; doi:10.1371/journal.pone.0245500)
Supplement: Attachment — Submitted filename: Review PONE_20_19005_R1.docx [file pone.0245500.s006.docx]

Review comments PONE D2019005R1

I thank the authors for attempting to resolve the outstanding issues with this important work. However major challenges remain mostly a valid reanalysis of the work for it to provide useful information to current practice/ care in the region.

1.	Findings based on this study are for a finite unrepresentative highly selected population to which inferences will be made given the nature of population used in this study. There is a high likelihood of unequal selection based on exposures/predictors and on whether one had a creatinine result or was alive or not lost to follow-up. It is stated that those excluded were similar to those included based on Age and sex etc, but nothing is stated in terms of WHO stage i.e. all clinical parameters so that we are confident that the presented findings could be representative of the target population. Also since this is a cohort being followed for change in renal function, we cannot divorce ourselves from the challenges related to cohort follow-up and these include: Loss to follow-up and competing risks in this case death prior to next creatinine. At a minimum these must be reported and how they were dealt with. Also the study population cannot be selected based on having or not having an outcome measurement otherwise we have unequal selection probability affecting participation hence selection bias. Also there is nothing in the paper to ascertain whether having or not having a creatinine was random or non-random.

2.	A wrong analysis was used—This study addresses the question of what the impact of ART use is on renal function within the context of an observational study. The question is how does use of ART change renal function overtime? First creatinine is a continuous outcome that could have benefited from an analysis that looks at it in its native data form while also accounting for within and between subject variability i.e mixed effects linear regression or Generalized Estimating Equations (GEE). Dichotomizing/categorizing and then using paired T-Tests and McNemar tests are acceptable with clinical trials but with this non-experimental study design leaves results from this sort of analysis confounded and hence biased. Logistic regression was used in the multivariate analysis doesn’t account for the correlated/dependent nature of the creatinine outcome measurements. Options are GEE or Generalized Linear mixed models (GLMM) analyses.

3.	There is unmeasured confounding--A common cause for ART regimen changes/use that could also affect renal function are various opportunistic infections as a results of clinical failure as well as there related treatments. These OIs are still not measured as stated by the authors. Also nothing about this bias is stated regarding their potential impact on the findings even in the limitations.

4.	Residual confounding--Also while OIs most of which are WHO stage III or IV conditions are not measured these could partially have adjusted for with WHO stage in the regression models. The current models don’t account for this hence the current estimates have residual confounding. Can the authors address this in their analysis. This is one of the flaws of backward selection since it doesn't account for empirical inherent biologically plausible relationships in the data for face/ content validity of the models.

5. Reverse causality--Are the predictors being reported predictors of ART use on renal function or predictors of survival with poor renal function and ART use? With the current design while the outcome of interest is incident (new ) renal failure after ART start, the authors actually insist on looking at prevalence at two time points baseline and sometime after ART start i.e. disease burden at two time points hence the difference in prevalence being attributed to ART use. Also patients with prevalent renal function at baseline are followed to this second time point. In this sort of analysis since you have prevalent disease at baseline being followed to the next time point we are unclear whether predictors are for survival or are causal. In fact given that a patient has baseline and a repeat creatinine suggests that they might have had some clinical sine qua non for poor renal function i.e. How sure are we that these patients were not likely to have clinical symptoms that require follow-up for renal impairment hence findings suggesting a high and uncommon 50% prevalence after 3 months on ART.
